# A Bimetallic Organic Framework with Mn in MIL-101(Cr) for Lithium–Sulfur Batteries

**DOI:** 10.3390/ma16103794

**Published:** 2023-05-17

**Authors:** Shuo Chen, Zhengfu Zhang, Jinsong Wang, Peng Dong

**Affiliations:** 1 Faculty of Materials Science and Engineering, Kunming University of Science and Technology, Kunming 650093, China; chenshuo970420@163.com (S.C.);; 2 Faculty of Metallurgical and Energy Engineering, Kunming University of Science and Technology, Kunming 650093, China

**Keywords:** lithium–sulfur batteries, pyrolysis, sulfur cathode

## Abstract

Lithium–sulfur batteries (LSBs) show excellent performance in terms of specific capacity and energy density. However, the cyclic stability of LSBs is compromised due to the “shuttle effect”, which hinders the practical applications of LSBs. Herein, a metal–organic framework (MOF) based on Cr ions as the main body composition, commonly known as MIL-101(Cr), was utilized to minimize the shuttle effect and improve the cyclic performance of LSBs. To obtain MOFs with a certain adsorption capacity for lithium polysulfide and a certain catalytic capacity, we propose an effective strategy of incorporating sulfur-loving metal ions (Mn) into the skeleton to enhance the reaction kinetics at the electrode. Based on the oxidation doping method, Mn^2+^ was uniformly dispersed in MIL-101(Cr) to produce bimetallic Cr_2_O_3_/MnO_x_ as a novel sulfur-carrying cathode material. Then, a sulfur injection process was carried out by melt diffusion to obtain the sulfur-containing Cr_2_O_3_/MnO_x_-S electrode. Moreover, an LSB assembled with Cr_2_O_3_/MnO_x_-S showed improved first-cycle discharge (1285 mAh·g^−1^ at 0.1 C) and cyclic performance (721 mAh·g^−1^ at 0.1 C after 100 cycles), and the overall performance was much better than that of monometallic MIL-101(Cr) as a sulfur carrier. These results revealed that the physical immobilization method of MIL-101(Cr) positively affected the adsorption of polysulfides, while the bimetallic composite Cr_2_O_3_/MnO_x_ formed by the doping of sulfur-loving Mn^2+^ into the porous MOF produced a good catalytic effect during LSB charging. This research provides a novel approach for preparing efficient sulfur-containing materials for LSBs.

## 1. Introduction

The energy storage technology revolution is in full swing. Lithium–sulfur batteries (LSBs) have a high energy density (2600 W·h·kg^−1^) [1], which demonstrates their strong potential in new energy storage applications [2,3,4]. However, the inferior cyclic stability of LSBs has hindered their practical application and commercialization. The shuttle effect and high potential energy barrier of the reaction when lithium polysulfides (Li_2_S*_x_*, 4 ≤ *x* ≤ 8) are reconverted into sulfur monomers (S_8_) significantly contribute to the poor cyclic stability of LSBs [5,6,7]. The shuttle effect occurs when the soluble lithium polysulfide generated at the positive electrode during the electrode reaction of LSBs is transferred with electrons to the negative electrode and directly reacts with the negative lithium electrode, resulting in an irreversible loss of active material in the battery. Hence, the cyclic capacity of LSBs quickly decays [8,9,10].

Sulfur/carbon (S/C) composites have been a popular cathode material for LSBs. The carbon material is mostly porous carbon, which can solve the problem of poor electrical conductivity in sulfur cathodes, and the porous carbon material usually has some pore structure to physically adsorb Li_2_S_x_, which can enhance the utilization of sulfur in LSBs. Although the addition of carbon materials allows for some improvement in the electrochemical properties of S/C composites, their cycling efficiency is not generally satisfactory, most notably due to the lack of active sites which do not adsorb Li_2_S_x_ strongly enough and still result in the loss of large amounts of Li_2_S_x_, eventually leading to a rapid decrease in the specific capacity of LSBs.

Significant effort has been made in the past few years to reduce the shuttle effect. The incorporation of metal–organic frameworks (MOFs) is one of the more promising solutions for solving this challenge [11,12,13,14]. MOFs represent a kind of organic–inorganic porous material comprising metal ions and organic ligands with ligand bonding. MOFs possess an ultra-high specific surface area and void fraction, and they show relatively good physical adsorption capacities for lithium polysulfides [15,16]. MOFs have more metal nodes (Lewis acidic sites) and organic functional groups than conventional porous carbon materials, which can provide binding sites for Li_2_S*_x_*. Moreover, these binding sites can confine lithium polysulfides to the pore sites of MOFs by physical adsorption [17,18,19]. Among the various types of MOFs, Lavoisier skeleton materials possess a large pore size and excellent stability. MIL-101(Cr) is an example of a Lavoisier skeleton material. Furthermore, MIL-101(Cr) possesses certain catalytic properties due to the presence of Cr [20,21]. It should be noted that high catalytic activity can increase the activation energy of chemical reactions, thereby reducing the effect of potential energy barriers in the reaction process [9]. Both Mn and Cr have exhibited excellent catalytic performance in different cells [9,22] and synergistic interactions between Cr and Mn oxides due to electron migration can lead to enhanced catalytic activity [23]. However, although MIL-101(Cr) performs well when used as a sulfur-containing cathode, certain problems still need to be solved. MOFs, including MIL-101(Cr), have poor conductivity [24], which affects their electrochemical performance. For the issue of insufficient electrical conductivity of MOF materials, some researchers have used pyrolysis to increase the electrical conductivity of MOF materials and it has worked well [25].

The current work focuses on the preparation of bimetallic MOFs using Mn-doped MIL-101(Cr) as a sulfur-containing cathode. The combination of Mn with Cr in the MIL-101(Cr) material provides a synergistic effect that improves its catalytic properties and maintains the MOF framework structure after pyrolysis. Therefore, the loading of S in the final electrode material does not decrease. MIL-101(Cr) was prepared by a hydrothermal method. This was followed by the preparation of Cr-Mn complexes, which were synthesized by combining manganese oxide with MIL-101(Cr), which has good catalytic oxidation activity and a high specific surface area. The content of Mn in the Cr-Mn complexes was the only factor that was changed, and the pyrolysis temperature was fixed. Finally, an electrode material was prepared by the melt-diffusion method. The current study investigated the structure of the composite Cr_2_O_3_/MnO_x_ material and its electrochemical performance as a cathode in LSBs.

## 2. Experimental Section

### 2.1. Sample Preparation

#### 2.1.1. Prepare of MIL-101(Cr) Precursor

The MIL-101(Cr) precursor was prepared using a hydrothermal method. Typically, 4.8 g of Cr(NO_3_)_3_·9H_2_O and 1.996 g of terephthalic acid (TPA) were mixed and ground. Then, the mixture was added to deionized water and stirred for 25 min. Next, 225 μL HF was added, and stirring was continued for a few minutes. This mixture was ultrasonically dispersed for 10 min, then placed in a polytetrafluoroethylene-lined reactor. The reactor was held at 220 °C for 15 h. A light green solid was obtained after the reaction. This solid was filtered and allowed to stand at 120 °C for 12 h to precipitate out the terephthalic acid vacancies. Finally, the powder was alternately washed 3 times with *N*,*N*-dimethylformamide (DMF), ethanol, and ultrapure water, then dried in a vacuum oven at 60 °C for 12 h. The light green powder obtained was the precursor of this research.

#### 2.1.2. Preparation of Cr_2_O_3_/MnO_x_ Composites

A total of 500 mg precursor was added to 80 mL ultrapure water and thoroughly stirred. Then, a certain mass of KMnO_4_ (53 mg, 1334 mg, or 2556 mg) was added into the stirred suspension, which was stirred for a further 2 h to prepare Cr-Mn composite materials with Mn loadings of 5 wt%, 10 wt% and 20 wt%. These were labeled as MIL-101-5Mn, MIL-101-10Mn and MIL-101-20Mn, respectively. Next, 4 mL of H_2_O_2_ (volume fraction of 30%) was diluted in 50 mL ultrapure water and stirred for 2 h. The stirred solution was washed three times with deionized water to obtain a dark green solid, which was denoted as MIL-101-Mn. The solid was heated at 900 °C at a heating rate of 5 °C/s in a protective atmosphere and pyrolysed for 2 h. The resulting dark green solid was denoted Cr_2_O_3_/MnO_x_.

#### 2.1.3. Preparation of MIL-101-S and Cr_2_O_3_/MnO_x_-S Electrode Materials

MIL-101(Cr) and Cr_2_O_3_/MnO_x_ were mixed with sulfur powder in a mass ratio of 3:7 using a grinding tool. Then, the mixed powders were homogeneously mixed by grinding for 30 min. After grinding, each powder was placed in an inert-atmosphere reactor and heated at 155 °C for 18 h. The dark green solid was obtained after cooling and denoted as MIL-101-S or Cr_2_O_3_/MnO_x_-S.

#### 2.1.4. Preparation of Batteries

To prepare the LSBs, super carbon black (super P) was applied as the conductive agent, polyvinylidene fluoride (PVDF) was applied as the binder, N-methyl-2-pyrrolidone (NMP) was applied as the solvent, and aluminum foil was used as the collector. The positive electrode of the battery was prepared using the active material after the sulfur loading with an active material: super P:PVDF ratio of 8:1:1. These components were added to a small bottle with NMP and stirred for 12 h. The mixed liquid was then evenly applied to the aluminum foil, which was dried in a vacuum oven at 70 °C for 12 h. After drying, the positive electrode was obtained. CR2025 button cells were used, and the electrolyte was 1 M LiTFSI (1,3-dioxolane: 1,2-dimethoxyethane = 1:1 *v*/*v*) + 1% LiNO_3_. The anode was lithium metal and the separator system was Polyethylene (Cyber Electrochemistry Materials, thickness: 25 μm, porosity: 45%). The corresponding positive electrode pieces were separately assembled into cells; the amount of electrolyte used in each cell was 20 μL.

### 2.2. Material Characterization

XRD analysis was performed with a Bruker D8 Advance (Billerica, MA, USA). Nitrogen adsorption–desorption analysis was concluded with a Micromeritics ASAP 2460 (Norcross, GA, USA) to determine BET surface areas and pore sizes. SEM and EDS analyses were performed with a ZEISS Gemini SEM 300 (Oberkochen, Germany), and TEM analysis was performed with a JEOL F200 (Tokyo, Japan). XPS was conducted with a Thermo Scientific K-Alpha spectrometer (Waltham, MA, USA) to investigate the elemental composition and valence states. The wavelengths of X-ray sources: XRD (Cu Kα1 = 0.15406 nm) and XPS (Al Kα1 = 0.8339 nm).

### 2.3. Electrochemical Characterization

The batteries were tested using a battery test system (SenveBTS, 5 V, 20 mA). The main current rate was 0.1 C (1 C = 1675 mA·g^−1^) and testing was carried out in the voltage range of 1.7 to 2.8 V. Cyclic voltammetry was carried out using a CHI760E workstation (Shanghai, China) in the voltage range of 1.6 to 3.0 V at a 1 mV·s^−1^ scanning rate.

## 3. Results and Discussion

### 3.1. Material Characterization

**Figure 1:**
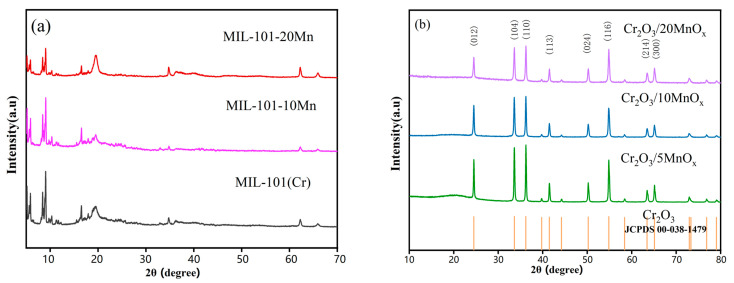
XRD spectra of (**a**) MOF precursor and MIL-101-10Mn material; (**b**) Cr_2_O_3_/MnO_x_ after pyrolysis.

**Figure 2:**
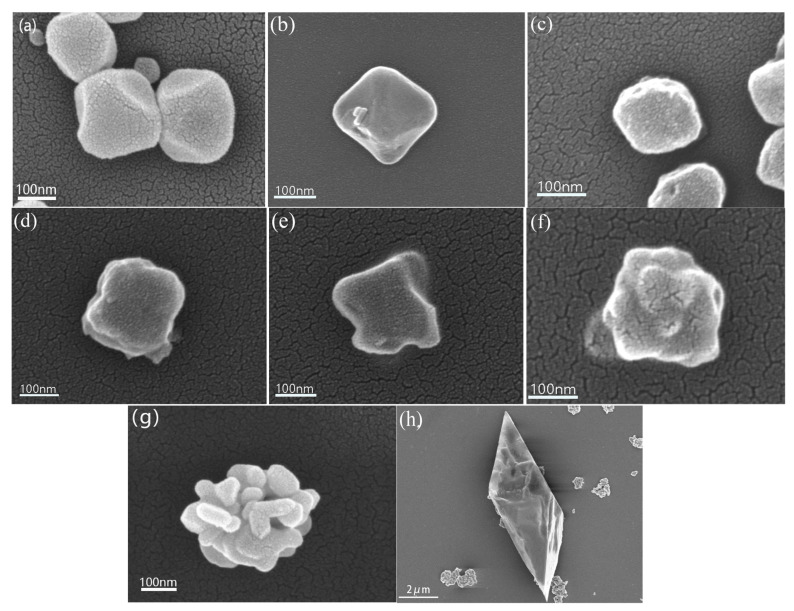
SEM photos of (**a**) MIL-101(Cr), (**b**) MIL-101-5Mn, (**c**) MIL-101-10Mn, (**d**) MIL-101-20Mn, (**e**) Cr_2_O_3_/5MnO_x_, (**f**) Cr_2_O_3_/10MnO_x_, (**g**) Cr_2_O_3_/20MnO_x_, and (**h**) Cr_2_O_3_/10MnO_x_-S.

Characterization of each material was carried out. MIL-101-Mn is a composite made of Cr-MOF loaded with different proportions of Mn, and Cr_2_O_3_/MnO_x_ is the MIL-101-Mn material after pyrolysis. Figure 1a shows the XRD patterns before pyrolysis. The XRD pattern of MIL-101(Cr) was almost identical to that of a previously reported work [26,27]. The peaks of the MIL-101(Cr) spectrum remained almost unchanged after Mn loading; meanwhile, the diffraction peaks at 19.3° and 37.6° were weakened due to the better dispersion of Mn. This demonstrates that the crystal structure of MIL-101(Cr) was not damaged during the loading process. However, the crystallinity was slightly lower, as demonstrated by the SEM images (Figure 2b–d). Figure 1b shows the XRD profiles of the Cr_2_O_3_/MnO_x_ samples after heat treatment, which are in agreement with the standard Cr_2_O_3_ diffraction peaks (JCPDS no. 00-038-1479). This demonstrates that the main form of Cr in Cr_2_O_3_/MnO_x_ after heat treatment was its oxide. Moreover, these diffraction patterns show that the dispersion of Mn was good for all loading ratios of MIL-101(Cr). No significant peaks related to Mn were observed in the XRD patterns because of the low Mn content and its existence in the amorphous state [28]. Table 1 shows the lattice parameters of each sample after heat treatment, and there was no significant change in the lattice parameters of the material as the Mn loading increased.

Figure 2a shows a regular ortho-octahedral structure, which is the microscopic morphology of MIL-101(Cr). Figure 2b–d show SEM images of MIL-101-nMn, demonstrating that the ortho-octahedral structure was maintained after Mn loading without any obvious collapse of the structural skeleton. Figure 2e–g show SEM images of MIL-101-nMn after heat treatment. Figure 2e shows that the particle size of Cr_2_O_3_/5MnO_x_ became smaller after pyrolysis. This is because MnO*_x_* entered the apertures of MIL-101(Cr) after loading, and a tight bond was created after heat treatment. Moreover, the particles became slightly rougher after heat treatment, indicating a minimal amount of structural collapse. Figure 2f,g show that the surface of the particles became rougher with increased MnO*_x_* loading, demonstrating that the MOF skeleton collapsed to a certain extent after heat treatment and that the Cr_2_O_3_/20MnO_x_ particles were deformed. Meanwhile, as MIL-101(Cr) started to decompose at 550 °C [29], the 900 °C pyrolysis used in this research caused the composite to decompose and the decomposed material was nucleated on top of the original material, which also caused the Cr_2_O_3_/MnO_x_ material to show a change in crystal particle size. Figure 2h shows the surface morphology of Cr_2_O_3_/10MnO_x_-S. The overall morphology of this material was still ortho-octahedral after 70 wt% sulfur loading.

**Figure 3:**
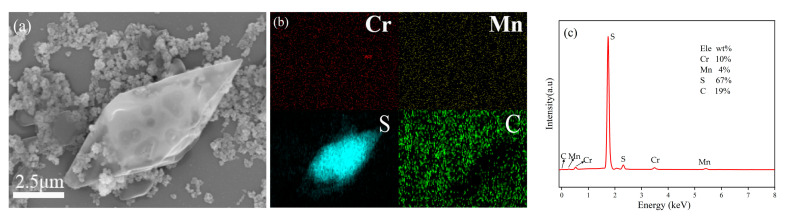
(**a**) SEM image, (**b**) elemental mapping, and (**c**) EDS image of Cr_2_O_3_/10MnO_x_-S.

Figure 3 shows the distribution of elements and EDS mapping of Cr_2_O_3_/10MnO_x_-S. As displayed in Figure 3a,b, S and Mn were very uniformly distributed, without any obvious sulfur agglomeration. The approximate content of each element in Cr_2_O_3_/10MnO_x_-S is shown in the energy spectrum displayed in Figure 3c. The elemental S content was approximately 67%, demonstrating the successful loading of S on Cr_2_O_3_/10MnO_x_.

**Figure 4:**
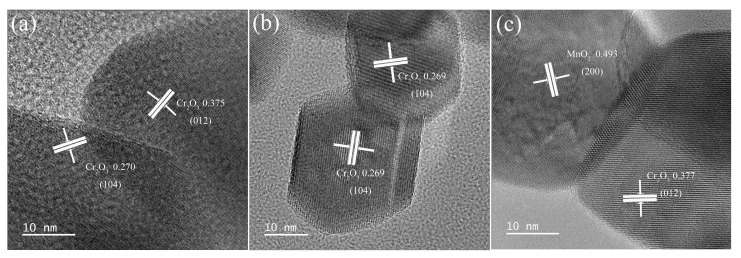
TEM images of (**a**) Cr_2_O_3_/5MnO_x_, (**b**) Cr_2_O_3_/10MnO_x_, and (**c**) Cr_2_O_3_/20MnO_x_.

Figure 4a–c show TEM images of the Cr_2_O_3_/MnO_x_ material, which exhibited lattice stripes ascribed to Cr_2_O_3_. The d-spacings of 0.375 nm and 0.270 nm correspond to the (012) and (104) planes of Cr_2_O_3_, respectively. These planes were also observed in the XRD patterns at 2θ = 24.5° and 33.5°, respectively. Moreover, a *d*-spacing of 0.494 nm was observed in the Cr_2_O_3_/10MnO_x_ and Cr_2_O_3_/20MnO_x_ samples, corresponding to the crystal surface of MnO*_x_*. This d-spacing corresponds to the (200) plane of α-MnO_2_ (JCPDS no. 00-044-0141) [30], which is in good accordance with the small surface particles observed in the SEM images of Cr_2_O_3_/MnO_x_. This also indicates that, when MnO*_x_* loading was increased to >10%, some of the MnO*_x_* was encapsulated on the MOF surface in the form of α-MnO_2_. Moreover, some short and intricate lattices were visible in the TEM images. These lattices exposed some of the high-energy crystal surfaces of Cr_2_O_3_. This phenomenon reduces the activation energy required for the conversion of lithium polysulfide to sulfur monomers.

**Figure 5:**
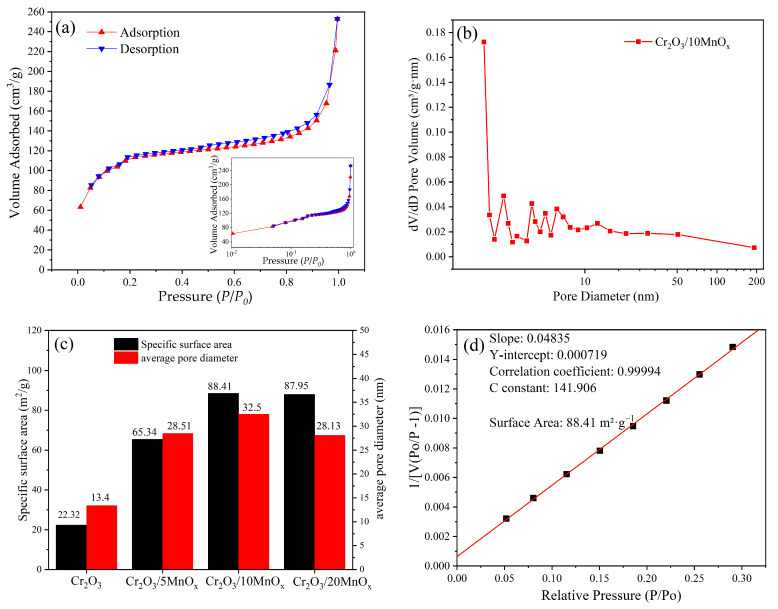
(**a**) N_2_ adsorption isotherm of Cr_2_O_3_/10MnO_x_ and extended hysteresis loops; (**b**) BJH pore size distribution of Cr_2_O_3_/10MnO_x_; (**c**) specific surface areas and average pore diameters of Cr_2_O_3_ and as-prepared Cr_2_O_3_/MnO_x_; (**d**) BET surface area plot of Cr_2_O_3_/10MnO_x_ at standard conditions for temperature and pressure (STP).

Figure 5a displays the N_2_ adsorption isotherm of Cr_2_O_3_/10MnO_x_. The Cr_2_O_3_/10MnO_x_ samples exhibited obvious type-IV adsorption isotherms with H_3_-type hysteresis loops, indicating the existence of a significant number of irregular mesopores [31]. The pore size distribution of Cr_2_O_3_/10MnO_x_ is shown in Figure 5b which demonstrated the existence of a great number of microporous and mesoporous constructions that can provide binding sites for the sulfur in the cell and for the polysulfides during the reaction. Figure 4c shows the specific surface areas (SSAs) and mean pore sizes of these materials. Because the Cr_2_O_3_/MnO_x_ composite is similar to Cr_2_O_3_, commercially available Cr_2_O_3_ (Shanghai Macklin Biochemical Co., Ltd.) was used for comparison. The SSAs and pore sizes of the as-fabricated composites were significantly higher than those of ordinary Cr_2_O_3_. The BET SSA of Cr_2_O_3_/5MnO_x_ was 65.34 m^2^·g^−1^ with a Barrett–Joyner–Halenda (BJH) average pore diameter of 28.51 nm; the BET SSA of Cr_2_O_3_/10MnO_x_ was 88.41 m^2^·g^−1^ with a BJH average pore diameter of 32.5 nm; and the BET SSA of Cr_2_O_3_/20MnO_x_ was 87.95 m^2^·g^−1^ with a BJH average pore diameter of 28.13 nm. Cr_2_O_3_/20MnO_x_ exhibited the highest SSA and Cr_2_O_3_/10MnO_x_ had the largest average pore diameter. The large SSA and small pore diameter of Cr_2_O_3_/20MnO_x_ was attributed to a large amount of Mn entering the apertures of MIL-101(Cr) to support the skeleton structure during pyrolysis and prevent excessive shrinkage; however, the excessive loading of MnO*_x_* decreased the average pore diameter.

**Figure 6:**
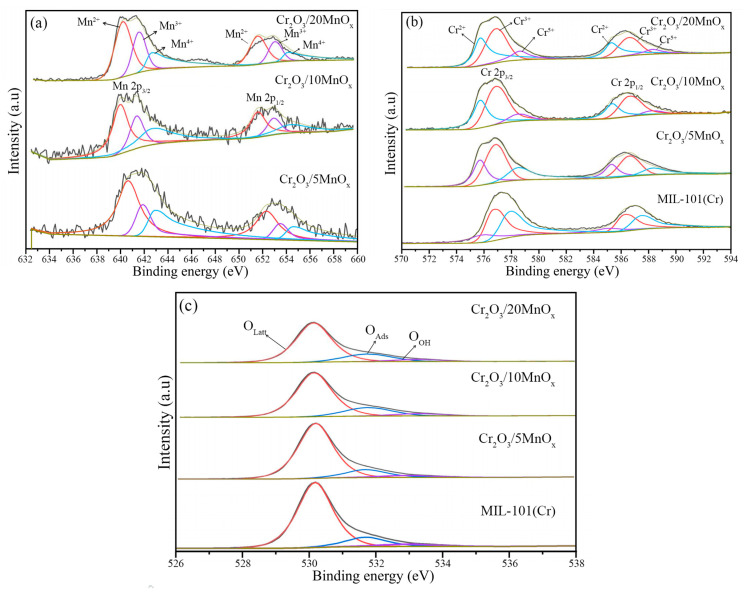
High-resolution (**a**) Mn 2p (Mn^2+^ is the red line, Mn^3+^ is the purple line, Mn^4+^ is the blue line), (**b**) Cr 2p (Cr^2+^ is the blue line, Cr^3+^ is the red line, Cr^5+^ is the purple line), and (**c**) O 1s (O_Latt_ is the red line, O_Ads_ is the blue line, O_OH_ is the purple line) spectra of the as-prepared MOFs.

Figure 6 shows the XPS spectra of the prepared samples, and the XPS-derived elemental composition data are summarized in Table 2. The high-resolution Mn 2p spectra of each sample are shown in Figure 6a. The main signals of Mn 2p_3/2_ were 640.01 eV(Mn^2+^), 641.38 eV(Mn^3+^) and 642.48 eV(Mn^4+^), while for Mn 2p_1/2_ the main signals were 652.22 eV(Mn^2+^), 653.67 eV(Mn^3+^) and 654.47 eV(Mn^4+^) [32,33]. Based on the data in Table 1, the Mn^3+^ fraction of Cr_2_O_3_/5MnO_x_ was lower than that of the other samples. The Cr 2p spectra of the samples are shown in Figure 6b. The main signals of Cr 2p_3/2_ were 575.42 eV(Cr^2+^), 576.57 eV(Cr^3+^) and 578.34 eV(Cr^4+^), while for Cr 2p_1/2_ the main signals were 585.21 eV(Cr^2+^), 586.47 eV(Cr^3+^) and 588.10 eV(Cr^4+^) [34,35,36,37]. A significant decline in Cr^5+^ content occurred after Mn loading, which, in combination with the presence and change in the valence state of Mn, can be attributed to electron migration between Mn and Cr: 2Mn^2+^ + Cr^5+^ ⇄ 2Mn^3+^ + Cr^3+^. Electronic migration between different metallic ions facilitates faster reaction rates and improves the catalytic ability of materials [38]. The O 1s spectra are shown in Figure 6c. The peak at 530.05 eV corresponds to lattice oxygen (O_Latt_), the peak at 531.70 eV corresponds to adsorbed oxygen (O_Ads_), and the peak at 533.22 eV relates to surface hydroxyl oxygen (O_OH_) [39,40]. The lattice oxygen content of Cr_2_O_3_/MnO_x_ was higher than that of MIL-101 (Cr). This promoted the oxidation process and enabled a certain oxygen spillover effect between MnO*_x_* and Cr_2_O_3_ after pyrolysis. Hence, lattice oxygen can readily migrate to the composite surface, thereby improving the catalytic efficiency of the Cr_2_O_3_/MnO_x_ composite.

### 3.2. Electrochemical Characterization

**Figure 7:**
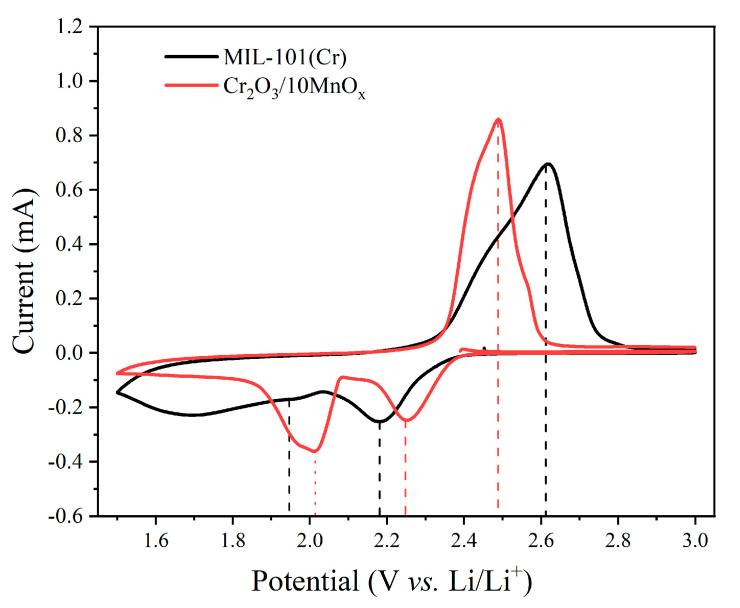
CV curves of MIL-101-S and Cr_2_O_3_/10MnO_x_-S at 1 mV·s^−1^.

The electrochemical properties of MIL-101-S and Cr_2_O_3_/MnO_x_-S were analyzed to evaluate the immobilization of sulfur in the MOF and the effect of adding Mn to MIL-101(Cr) on the specific capacity and cycling stability. The mass loading of sulfur for all electrode materials was 1.6 mg·cm^−2^. Figure 7 displays the cyclic voltammetry (CV) profiles of Cr_2_O_3_/10MnO_x_-S, which displayed clear reduction peaks at 2.23 and 2.02 V due to the conversion of S_8_ molecules to higher-order Li_2_S_x_ and their further conversion to Li_2_S_2_ and Li_2_S during the discharge process. The oxidation peak at 2.45 V corresponds to the conversion of polysulfides into S_8_ molecules (Equations (1) and (2)), as follows:

*x*S_8_ + 16e^−^ + 16Li^+^ ⇆ 8Li_2_S*_x_* (4 ≤ *x* ≤ 8) (1)

4Li_2_S*_x_* + (6*x* − 8)e^−^ + (6*x* − 8)Li^+^ ⇆ *x*Li_2_S_2_ + 2*x*Li_2_S (2)

The CV peaks further indicate that the chemical reactions are reversible [17,41]. It should be noted that the Cr_2_O_3_/10MnO_x_-S electrode had a higher reduction peak and a lower oxidation peak than the MIL-101-S electrode. In the corresponding charge/discharge profiles, the charge/discharge potential difference of the Cr_2_O_3_/10MnO_x_-S electrode was also smaller. Thus, the CV analysis shows that Mn loading caused the chemical reaction to proceed more smoothly, leading to higher catalytic activity and higher utilization of the active material. The electrochemical stability was further assessed by carrying out the charge/discharge process at 0.1 C.

**Figure 8:**
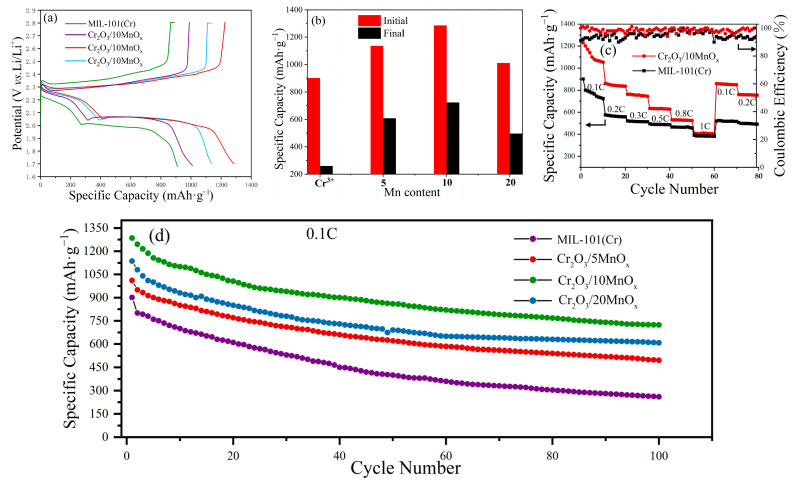
(**a**) Initial charge/discharge curves at 0.1 C; (**b**) specific capacity as a function of Mn loading; (**c**) cyclic performance of MIL-101-S, Cr_2_O_3_/5MnO_x_-S, Cr_2_O_3_/10MnO_x_-S, and Cr_2_O_3_/20MnO_x_-S electrodes at 0.1 C; (**d**) cyclic performance of MIL-101-S, Cr_2_O_3_/5MnO_x_-S, Cr_2_O_3_/10MnO_x_-S, and Cr_2_O_3_/20MnO_x_-S electrodes at 0.1 C.

Figure 8a shows that the initial discharge capacity of the MIL-101-based LSB was 901.8 mAh·g^−1^, and this capacity decreased to 261.3 mAh·g^−1^ after 100 cycles. Furthermore, similar to the CV curves, the galvanostatic charge/discharge curves of Cr_2_O_3_/MnO_x_-S exhibited two distinct plateaus at about 2.23 V and 2.02 V. The materials prepared with different Mn contents showed significant differences in terms of initial discharge performance. The curves in the figure show that the Cr_2_O_3_/MnO_x_-S electrodes with different Mn loadings all performed better than the original MOF material in terms of first discharge specific capacity, with Cr_2_O_3_/10MnO_x_-S having the highest specific capacity (1286 mAh·g^−1^). The specific capacity of Cr_2_O_3_/20MnO_x_-S became lower compared to Cr_2_O_3_/10MnO_x_-S, probably because the Mn loading had exceeded the upper limit, the material pores were over-occupied and the sulfur in the electrode material formed aggregates, leading to a lower utilization of the active material during the electrode reaction. MIL-101-S and Cr_2_O_3_/20MnO_x_-S exhibited different degrees of overcharging during the charging process. It should be noted that MIL-101-S does not contain any Mn. Hence, MnO_x_ crystals were not formed, resulting in a significant collapse of the MIL-101(Cr) channels during pyrolysis. Therefore, S molecules were not able to enter this MOF, leading to S agglomeration and reducing the utilization of sulfur during the initial discharge. The overcharging of Cr_2_O_3_/20MnO_x_-S was attributed to the excessive addition of Mn, which filled the MOF channels with MnO_x_. This reduced the ability of S to enter the structure and lowered the sulfur utilization. To show the difference in the performance of the Cr_2_O_3_/MnO_x_-S and MIL-101-S electrodes, Figure 8b shows the initial specific capacity of these materials and their specific capacity after 100 cycles at 0.1 C. The initial specific capacity of the Cr_2_O_3_/MnO_x_-based LSB and its specific capacity after cycling were better than those of MIL-101(Cr).

To assess their rate performance, the Cr_2_O_3_/10MnO_x_-S and MIL-101-S electrodes were cycled at different charge and discharge rates (0.1, 0.2, 0.3, 0.5, 0.8 and 1 C) [17]. After cycling, the electrodes were tested again at low current rates (0.1 C and 0.2 C) to assess their capacity recovery. The results are presented in Figure 8c. In general, a lower discharge rate corresponds to higher capacity and vice versa [42,43]. It should be noted that capacity declines at high discharge rates due to the limited free space for Li^+^ ions [17,44]. There is a relatively high potential energy barrier for the conversion of polysulfides into sulfur molecules during the charge/discharge reaction of LSBs, leading to inferior cyclic performance [45]. However, the addition of a catalyst can reduce the activation energy of these reactions and improve the cycling efficiency of LSBs [46]. The specific capacity of Cr_2_O_3_/10MnO_x_-S exceeded that of MIL-101-S at all discharge rates. In terms of overall cyclic efficiency, the addition of Mn improved the specific capacity and cyclic efficiency of the battery at low discharge rates, while only a limited improvement was observed at high discharge rates. This was because the addition of Mn led to the Mn occupying some of the pore volume of the MOF material, limiting the space for Li^+^ ions. Thus, the effect of the catalyst on the cyclic efficiency of the battery became insignificant. Meanwhile, Figure 8c shows that the Cr_2_O_3_/10MnO_x_-S and MIL-101-S electrodes behaved differently in terms of charging efficiency. The Cr_2_O_3_/10MnO_x_-S electrode maintained a charging efficiency of about 98%, but the MIL-101-S electrode exhibited a lower charging efficiency than Cr_2_O_3_/10MnO_x_-S, indicating a certain degree of electrochemical polarization. The poor electrical conductivity of the MIL-101-S electrode resulted in a low rate of electron conduction during the cell reaction. The Cr_2_O_3_/10MnO_x_-S material had enhanced electrical conductivity after pyrolysis and achieved higher catalytic activity after loading with Mn. Hence, at the same discharge rate, the electrode reaction of Cr_2_O_3_/MnO_x_-S was enhanced compared to that of the MIL-101-S electrode, resulting in a higher charging efficiency.

To fully demonstrate the overall improvement of the cyclic efficiency of the MOF after adding Mn addition, the MIL-101-S and Cr_2_O_3_/MnO_x_-S electrodes were tested for 100 cycles at 0.1 C, and the results are shown in Figure 8d. The performance of the MIL-101-S electrode was inferior to that of the Cr_2_O_3_/MnO_x_-S electrodes in terms of both initial discharge capacity and cyclic stability. The Cr_2_O_3_/5MnO_x_-S electrode had a slightly improved performance compared to the MIL-101-S electrode, while the optimal performance was achieved at a Mn loading of 10%. The Cr_2_O_3_/10MnO_x_-S electrode had an initial specific capacity of 1286 mAh·g^−1^ and a 100th cycle capacity of 721 mAh·g^−1^. At a Mn loading of 20%, the initial specific capacity decreased but the cyclic performance was slightly improved because the excessive loading of Mn lowered the average pore size (Figure 5b), resulting in sulfur aggregation and reduced sulfur utilization. Hence, the overall specific capacity of this electrode was lower.

## 4. Conclusions

In summary, MIL-101(Cr) was synthesized by a hydrothermal method. Cr_2_O_3_/MnO_x_ was then prepared by pre-impregnation, reduction and pyrolysis. The resulting Cr_2_O_3_/MnO_x_-S material was utilized as a cathode for lithium–sulfur batteries. The effects of Mn content on the morphology, structure, specific surface area, catalytic performance and electrochemical properties of this electrode were systematically investigated. The results revealed that, even with a small amount of Mn, the performance of the battery cathode was significantly improved. The morphology of the Cr_2_O_3_/MnO_x_ composite prepared after Mn loading was ortho-octahedral, where Cr_2_O_3_/10MnO_x_ possessed a BET SSA of 88.41 m^2^·g^−1^ and a BJH average pore diameter of 32.5 nm. The positive material Cr_2_O_3_/10MnO_x_-S possessed the best electrochemical performance with a sulfur mass loading of 1.6 mg·cm^−2^. Cr_2_O_3_/10MnO_x_-S achieved an initial discharge capacity of 1286 mAh·g^−1^ (0.1 C) and retained a capacity of 721 mAh·g^−1^ after 100 charge/discharge cycles.

## Figures and Tables

**Table 1:** Lattice parameters of the as-prepared MOFs.

Sample	a	b	c
Cr_2_O_3_/5MnO_x_	4.9569	4.9569	13.599
Cr_2_O_3_/10MnO_x_	4.9590	4.9590	13.589
Cr_2_O_3_/20MnO_x_	4.9623	4.9623	13.614

**Table 2:** XPS results of the as-prepared MOFs.

Sample	x(Mn^3+^)/x(Mn)	x(Mn^2+^)/x(Mn)	x(Cr^5+^)/x(Cr)	x(O_latt_)/x(O_Ads_)
MIL-101(Cr)	-	-	0.432	12.34
Cr_2_O_3_/5MnO_x_	0.278	0.503	0.382	23.37
Cr_2_O_3_/10MnO_x_	0.511	0.373	0.402	21.33
Cr_2_O_3_/20MnO_x_	0.454	0.412	0.402	25.88

## Data Availability

The data used to support the findings of this study are available from the corresponding author upon request.
